# Supplementary material for: Genetic comparisons of interleukin-17 reveal a framework for complex signaling evolution
Source: bioRxiv. 2026 Apr 14:2026.04.13.718218. Preprint. [Version 1] doi: 10.64898/2026.04.13.718218 (PMC13105070; doi:10.64898/2026.04.13.718218)
Supplement: Supplement 3 [file media-3.zip › Supplemental files for paper/ERC/IL17xIL17_comparisons/IL25_IL17F.pdf]

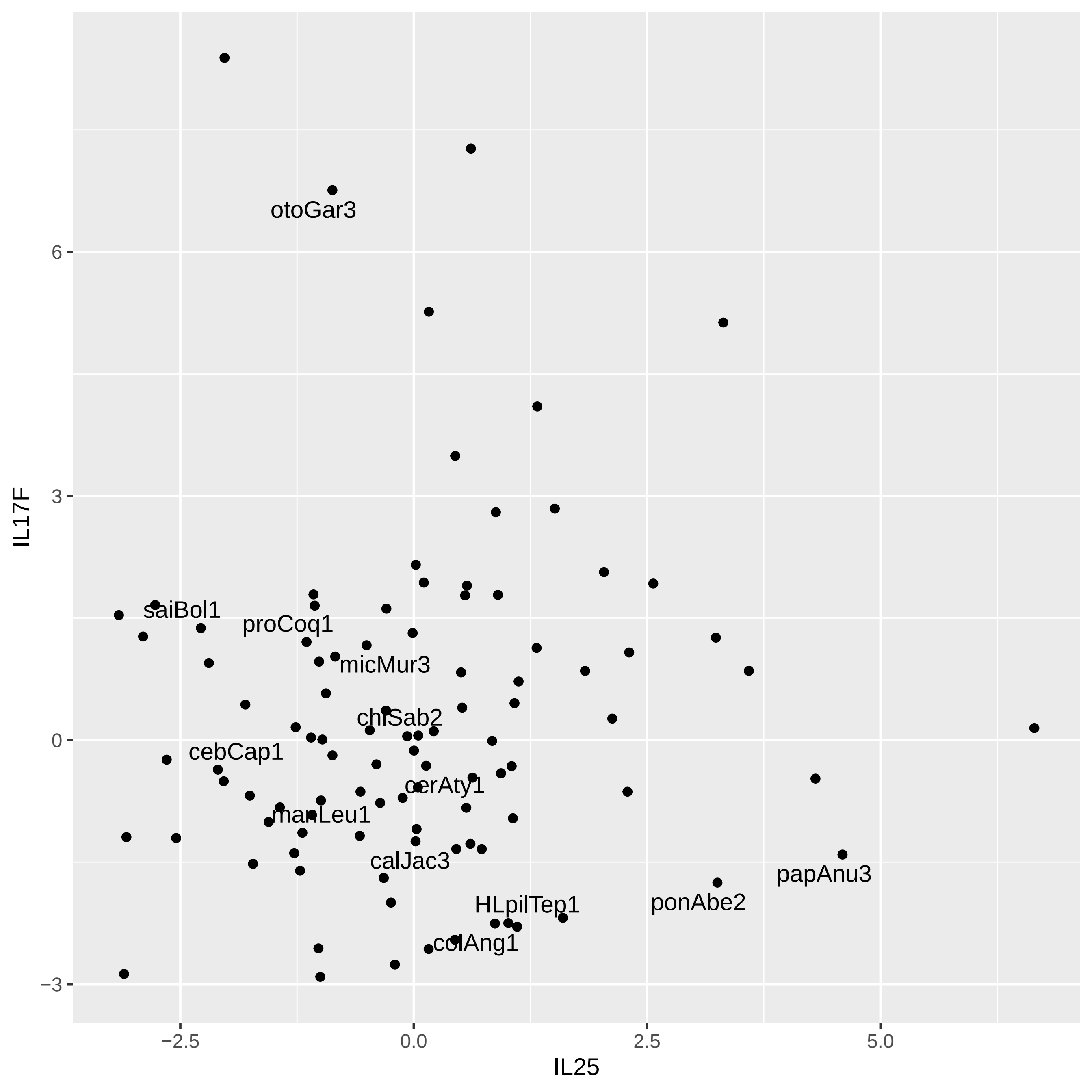

otoGar3
saiBol1
proCoq1
micMur3
chlSab2
cebCap1
cerAty1
manLeu1
calJac3
papAnu3
ponAbe2
HLpilTep1
colAng1
6
3
0
−3
−2.5
0.0
2.5
5.0
IL17F
IL25
